# DC8 and DC13 *var* Genes Associated with Severe Malaria Bind Avidly to Diverse Endothelial Cells

**DOI:** 10.1371/journal.ppat.1003430

**Published:** 2013-06-27

**Authors:** Marion Avril, Andrew J. Brazier, Martin Melcher, Sowmya Sampath, Joseph D. Smith

**Affiliations:** 1 Seattle Biomedical Research Institute, Seattle, Washington, United States of America; 2 Department of Global Health, University of Washington, Seattle, Washington, United States of America; University of Melbourne, Australia

## Abstract

During blood stage infection, *Plasmodium falciparum* infected erythrocytes (IE) bind to host blood vessels. This virulence determinant enables parasites to evade spleen-dependent killing mechanisms, but paradoxically in some cases may reduce parasite fitness by killing the host. Adhesion of infected erythrocytes is mediated by *P. falciparum* erythrocyte membrane protein 1 (PfEMP1), a family of polymorphic adhesion proteins encoded by *var* genes. Whereas cerebral binding and severe malaria are associated with parasites expressing DC8 and DC13 *var* genes, relatively little is known about the non-brain endothelial selection on severe malaria adhesive types. In this study, we selected *P. falciparum*-IEs on diverse endothelial cell types and demonstrate that DC8 and DC13 *var* genes were consistently among the major *var* transcripts selected on non-brain endothelial cells (lung, heart, bone marrow). To investigate the molecular basis for this avid endothelial binding activity, recombinant proteins were expressed from the predominant upregulated DC8 transcript, IT4var19. In-depth binding comparisons revealed that multiple extracellular domains from this protein bound brain and non-brain endothelial cells, and individual domains largely did not discriminate between different endothelial cell types. Additionally, we found that recombinant DC8 and DC13 CIDR1 domains exhibited a widespread endothelial binding activity and could compete for DC8-IE binding to brain endothelial cells, suggesting they may bind the same host receptor. Our findings provide new insights into the interaction of severe malaria adhesive types and host blood vessels and support the hypothesis that parasites causing severe malaria express PfEMP1 variants with a superior ability to adhere to diverse endothelial cell types, and may therefore endow these parasites with a growth and transmission advantage.

## Author Summary

The virulence of *P. falciparum* has been linked to the ability of infected erythrocytes to bind to receptors on the endothelial lining of blood vessels and sequester from blood circulation. This phenotype enables parasites to avoid spleen removal and is associated with organ-specific disease complications. This binding interaction is mediated by a large family of adhesion proteins, termed *P. falciparum* erythrocyte membrane 1 (PfEMP1), but relatively little is known about endothelial tissue selection and if there are parasite selective advantages to potentially deadly binding variants. In this study, we showed that two PfEMP1 subtypes that have been associated with severe malaria infections (DC8 and DC13) were among the best binders to a diversity of endothelial cell types. This avid endothelial binding phenotype was associated with PfEMP1 proteins that encoded multiple binding domains with widespread endothelial binding activity to brain, heart, lung, and bone marrow cells. These traits may give parasites expressing DC8 and DC13 PfEMP1 variants a growth and transmission advantage, even though sequestration in these tissues may result in deadly complications in a subset of hosts.

## Introduction

*Plasmodium falciparum* is a mosquito-borne infectious pathogen that is responsible for about 200–300 million illnesses and around 700,000 deaths each year [1]. A major virulence determinant for *P. falciparum* is the cytoadherence and sequestration of infected erythrocytes in capillaries and post-capillary venules of host organs, particularly the small intestine, heart, lung, and brain [2]–[4]. Cytoadhesion is mediated by the large and diverse *var* gene or *P. falciparum* erythrocyte membrane protein 1 (PfEMP1) family [5]–[7]. *Var* genes undergo transcriptional switching to modify the binding and antigenic properties of IEs [6], [8]. Although several endothelial binding properties have been mapped in PfEMP1 proteins [9], [10] relatively little is known about how PfEMP1s determine microvascular tropism.

Analysis of *var* gene sequences indicates that the family is evolving into separate groups defined by distinct upstream sequences, protein domain architectures, and protein binding properties [9], [11]–[15]. The majority of PfEMP1 proteins are classified into groups A, B, or C based on chromosome location and upstream sequence [12]–[14]. There is also a small subset of chimeric *var* genes, termed group B/A, and three unusual strain transcendent *var* genes (VAR1CSA, VAR2CSA, and Type 3 var) found in most or all parasite genotypes [14], [16]. PfEMP1 are large proteins that contain between two to nine adhesion domains termed Duffy binding-like (DBL) and cysteine-rich interdomain region (CIDR). Most proteins encode a semi-conserved head structure composed of an N-terminal DBL domain followed by a CIDR domain [7]. The CIDR1 domain in the PfEMP1 semi-conserved head structure plays a key role in IE binding. This domain is under strong selection to bind the host receptor CD36 in most group B and C PfEMP1 variants, but does not bind CD36 in the group A and B/A proteins [17], [18]. Significantly, group A and B/A proteins tend to be expressed in young children with limited malaria immunity and a subset of group A and B/A proteins termed DC8 and DC13 mediate cerebral binding and have been associated with severe malaria complications [19]–[26]. Similarly, the isolate-transcendent VAR2CSA variant is directly linked to pregnancy associated malaria [27]. Both the hierarchical expression of distinct PfEMP1 binding variants in African children's infections and the specialization of VAR2CSA for placental binding suggest that *var* groups are evolving for distinct binding and host niches.

The *var* gene family has ancient origins in a lineage of *P. falciparum*-like species that infect humans and chimpanzees [14], [16], [28]. The low frequency of serious clinical complications arising from malaria infections (∼2%) suggests the cytoadhesion traits have evolved to limit life-threatening reactions in the host, although IE cytoadhesion in brain and placenta are associated with severe disease. While placental malaria leads to low birth weight infants who are at greater risk of death, it generally does not kill pregnant women in higher malaria transmission areas [29]. Conversely, cerebral malaria is a major life-threatening complication that carries a 15–20% case fatality rate in hospital settings and kills hundreds of thousands of children each year [30]. Although DC8 and DC13 variants are not restricted to severe malaria infections [24], it is therefore surprising that *P. falciparum* retains a subset of PfEMP1 proteins, which might be expected to reduce parasite fitness by rapidly killing the host. The nature of the selective advantage that maintains these potentially deadly adhesion traits in the parasite population is unclear.

In order to investigate this seemingly paradoxical characteristic of DC8 and DC13 *var* genes, we developed an in vitro assay to investigate the endothelial selection on PfEMP1 proteins. This analysis revealed that DC8 and DC13 *var* genes were preferentially selected on diverse endothelial cell types and that multiple adhesion domains in DC8 variants bind host receptors with widespread endothelial distribution (brain, heart, lung, and bone marrow). This unexpected adhesion property could contribute to parasite fitness and at least partially explain why these severe malaria adhesion types tend to be expressed in malaria naïve hosts and have been retained in the parasite population.

## Results

### Panning of *P. falciparum*-IEs on diverse microvascular endothelial cell types selects for parasite binding variants that are not dependent on the common cytoadhesion receptors CD36 or ICAM1

**Figure 1:**
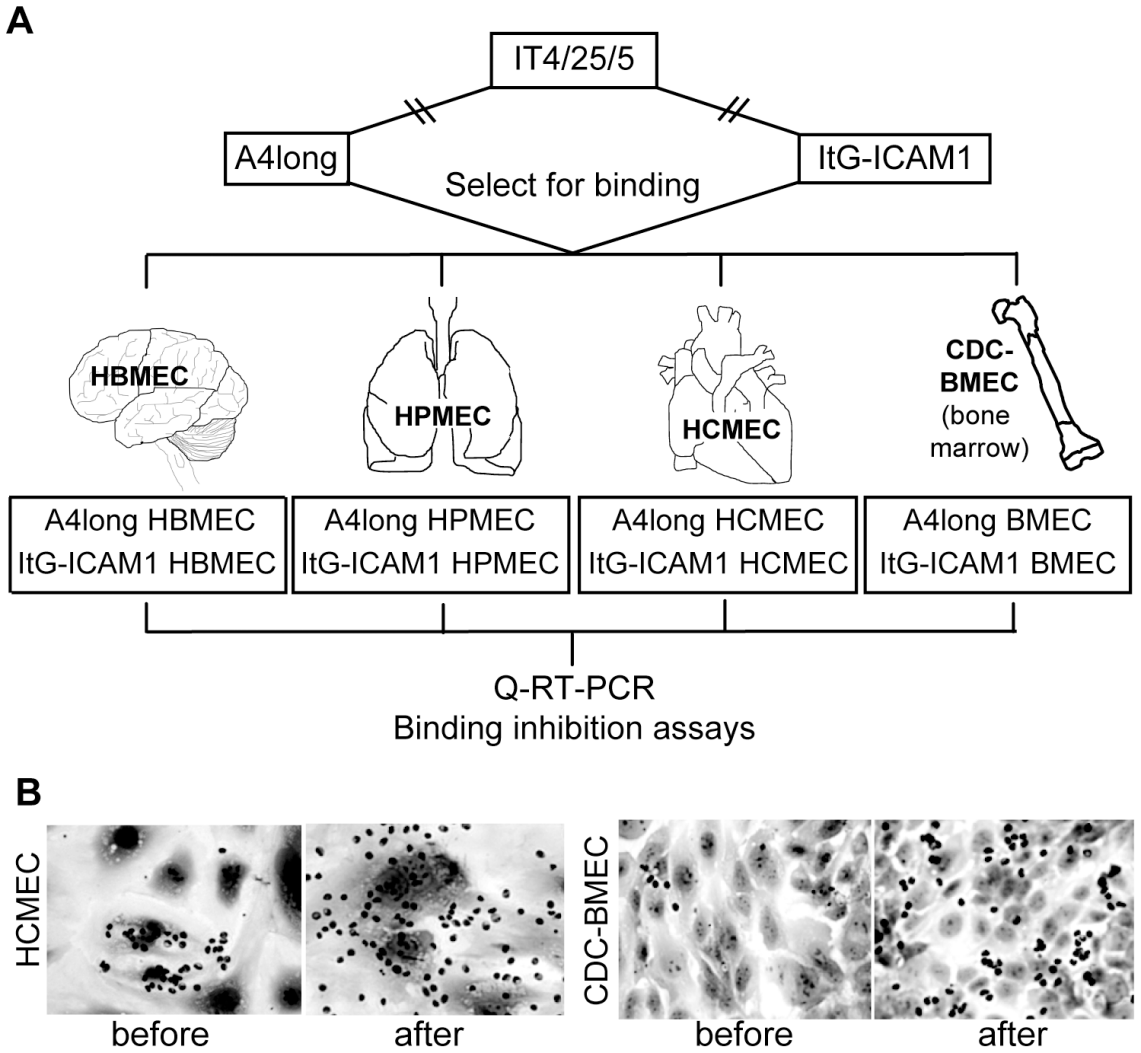
Selection of *P. falciparum*-IEs on different endothelial cell types. (A) A panel of six parasites lines was generated from the A4long and ItG-ICAM1 parasite lines by three rounds of selection on HPMEC (pulmonary), HCMEC (cardiac), and CDC-BMEC (bone marrow) cells. Selection on HBMEC (brain) was reported previously [19]. (B) IEs exhibited a concentrated binding pattern to a subpopulation of endothelial cells before selection and a more diffuse binding pattern on the entire population of cells after three rounds of selection.

Whereas CD36 and ICAM1 are the most common adhesion properties of *P. falciparum* infected erythrocytes [17], [18], [31], we previously found that non-CD36 binding DC8 *var* genes were selected on primary human brain endothelial cells [19]. Here we evaluated whether distinct *var* gene transcripts would be selected from the same two parasite lines when they were selected on non-brain endothelial cells (lung, heart, and bone marrow) (Figure 1). The A4long (CD36 binder and weak ICAM1 binder) and ItG-ICAM-1 (CD36 and ICAM-1 binder) parasite are both derived from the IT4/25/5 parental strain, but express different *var* genes [19].

Prior to *P. falciparum*-IE selection, endothelial cells were compared for surface receptor expression. All of the endothelial cells stained positively for the endothelial cell makers von Willebrand Factor (VWF) and PECAM1 (CD31) (Table S1 and Figure S1). In addition, we found that the rolling adhesion receptor ICAM1 [32]–[34] is highly expressed by over 90% of transformed human brain microvascular endothelial cells (THBMEC), human pulmonary microvascular endothelial cells (HPMEC), and human cardiac microvascular endothelial cells (HCMEC), but was expressed at much lower levels and on only 25% of bone marrow endothelial cells (CDC-BMEC) (Table S1, Figure S1). By comparison, the major cytoadhesion receptor CD36 [35], [36] was weakly expressed by most endothelial cells. HPMEC had the highest CD36 expression levels and greatest percentage of surface positive cells (33%), while CD36 was expressed on less than 5% of HCMEC, THBMEC, and CDC-BMEC (Table S1 and Figure S1). In addition, three other potential parasite cytoadhesion receptors [37], [38] were poorly expressed on resting endothelial cells. Vascular cell adhesion molecule-1 (VCAM1) was moderately expressed by THBMEC, but was weak or absent on other endothelial cells. Endothelial-leukocyte adhesion molecule-1 (ELAM1) and fractalkine (CX3CL1) were generally not expressed on any resting endothelial cells (Table S1, Figure S1). Lastly, THBMEC expressed little or no intercellular adhesion molecule-2 (ICAM2), whereas it was strongly expressed by HPMEC, HCMEC, and CDC-BMEC.

**Figure 2:**
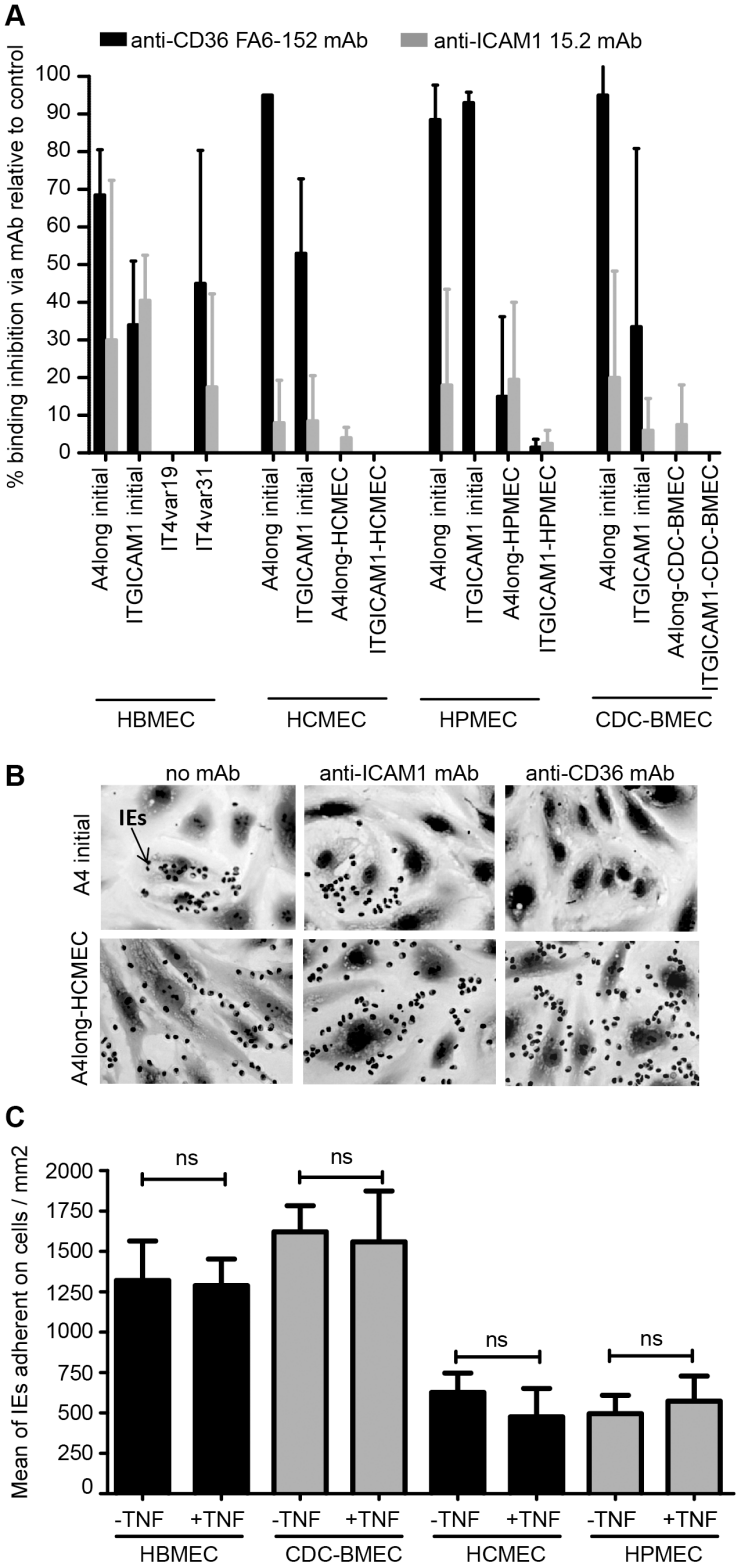
Interaction of *P. falciparum*-IEs with different endothelial cell types. (A) The ability of anti-CD36 (FA6-152) and anti-ICAM1 (15.2) monoclonal antibodies to block IE binding to different endothelial cell types was compared between starting and selected parasite lines. IT4var19 (DC8) and IT4var31 (CD36 binder) are cloned parasite lines that predominately transcribe a single *var* gene [19]. Binding results are expressed as percentage of inhibition relative to antibody-free controls. (B) Binding of starting and selected A4long parasite lines to HCMEC in the presence or absence of CD36 or ICAM1 monoclonal antibodies. Arrow points to a *P. falciparum*-IE (black dots in image). (C) *P. falciparum*-infected erythrocytes expressing the DC8 variant (IT4var19) were compared for binding to untreated or TNF-α activated endothelial cells.

Similar to primary human brain microvascular endothelial cells (HBMEC) [19], the starting A4long and ItG-ICAM-1 parasite lines adhered to only a minor subpopulation of non-brain endothelial cells in each case (Figure 1). The initial binding for both starting parasite lines was strongly dependent on CD36 (30–90% inhibition via anti-CD36 antibodies) and partially dependent on ICAM1 (10–40% inhibition with anti-ICAM1 antibodies) (Figure 2A–B). However, after panning the parasites three times on each of the non-brain endothelial cell lines, binding levels increased 3–10 fold for the different selected parasite lines (Figure S2) and the IEs exhibited a new dispersed binding phenotype on all of the different endothelial cell types (Figure 1B). In addition, all six sets of parasites selected on the non-brain endothelial cells had acquired a new binding phenotype and were no longer dependent on CD36 or ICAM1 for endothelial binding (Figure 2A). The only minor exception was the A4long parasite line selected on HPMEC cells, which was weakly inhibited (∼20%) by anti-CD36 and anti-ICAM1 antibodies. In summary, panning on non-brain endothelial cells selected for new parasite adhesion types that displayed a more dispersed binding phenotype and were not as dependent on CD36 or ICAM1 for adhesion.

### DC8 and DC13 *var* genes were consistently selected on different endothelial cell types

**Figure 3:**
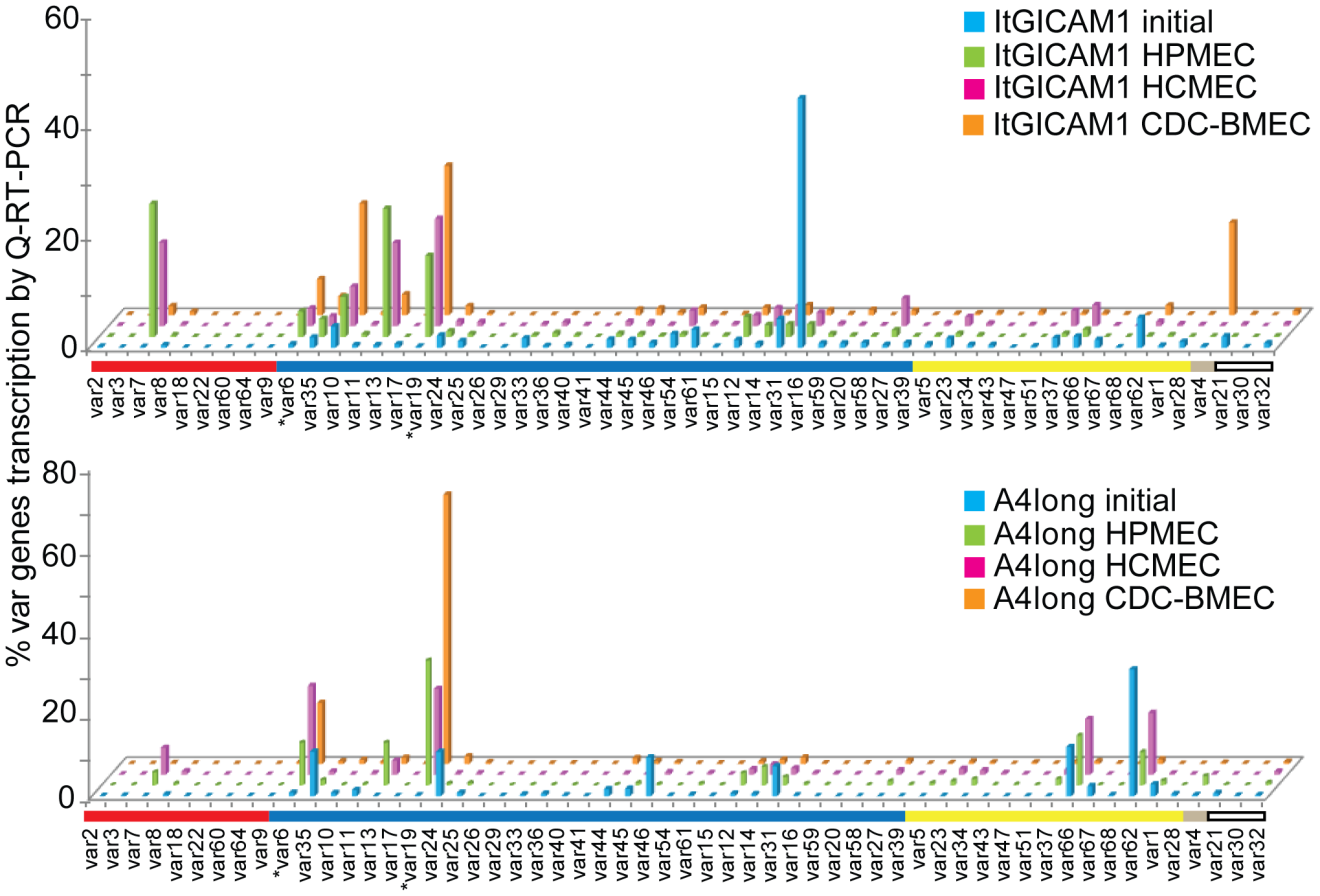
DC8 and DC13 *var* transcripts are consistently upregulated on different microvascular endothelial cell types. Transcription of *var* genes was analyzed by Q-RT-PCR from ring-stage IEs isolated before selection (blue) or after selection on HPMEC (green), HCMEC (pink), or CDC-BMEC (orange). Results were normalized to the housekeeping gene adenylosuccinate lyase (asl) and expressed as percentage of *var* genes transcribed relative to the total of 56 *var* genes analyzed. Genes are organized by Ups category; UpsA (red), UpsB (dark blue), UpsC (yellow), UpsE (grey), undetermined (white). Group B/A *var6* and *var19* are indicated with an asterisk.

To investigate if different endothelial cells selected for distinct *var* genes, the selected parasite lines were analyzed for *var* gene expression using quantitative (Q)-RT-PCR with a comprehensive array of primers encompassing the IT4 *var* gene repertoire. Our group and others previously found that DC8 and DC13 *var* genes were consistently selected from different parasite strains on brain endothelial cells [19], [21]. Similar to our previous experience with THBMEC panning, we found that the same two DC8 *var* genes (*IT4var6* and *IT4var19*) and a DC17 *var* gene (*IT4var13*) were strongly selected on all three endothelial cell types (Figure 3). In addition, panning on HPMEC or HCMEC upregulated the DC13 *var* gene (*IT4var7*) gene in both ItG-ICAM1 and A4long selected IEs. Parasites expressing *IT4var7* and *IT4var19* were also previously selected on a different immortalized brain endothelial cell line [21].

In addition to the 4 major *var* transcripts selected on all non-brain endothelial types, there were also some tissue-specific *var* transcripts. For instance, *IT4var62* and *IT4var66* (group C) were expressed by the starting A4long parasite line prior to selection and remained expressed at the same or a slightly reduced proportion of the total *var* transcripts in parasites selected on pulmonary and cardiac endothelial cells. However, they were down-selected in bone marrow endothelial cells and were not selected in the ItG-ICAM-1 parasite line. In addition, *IT4var4/var2CSA* (group E) was selected on bone marrow endothelial cells. Conversely, the starting *IT4var16* transcript in the ItG-ICAM-1 parasite line was strongly down-selected in all of the endothelial panned parasite lines (Figure 3), despite the fact that IT4var16 binds CD36 and ICAM-1 [39] and all of the endothelial cells were strongly surface positive for ICAM1, except for CDC-BMEC (Table S1).

To rule out the possibility that the parasites expressing DC8 and DC13 PfEMP1s were selected as a consequence of more rapid growth rather than more robust adhesion to the various endothelial cell types, we compared the growth rate of the starting ItG-ICAM-1 parasite line (ICAM-1 binder) to cloned parasite lines expressing one of the upregulated DC8 *var* (*IT4var19*) or a CD36 binding variant (*IT4var31*). All three parasite lines had similar growth rates over 4 cycles of parasite growth (Figure S3). We therefore concluded that the emergence of the parasites expressing DC8 and DC13 PfEMP1s after panning was attributable to the adhesion traits mediated by these proteins, rather than more rapid growth amongst parasites in which these *var* genes had been upregulated. On this basis we determined that the DC8 and DC13 *var* products, which do not encode CD36 or ICAM1 binding activity [19], were likely to be responsible for the new dispersed endothelial binding phenotype (Figure 2).

### DC8-IT4var19 encodes multiple domains with endothelial binding activity

To investigate the molecular mechanisms associated with this broad endothelial binding phenotype, we focused on one of the DC8 transcripts (*IT4var19*) that was consistently upregulated on the four endothelial cell types. TNF-α is produced during malaria infection [40] and upregulates adhesion molecules on the vascular endothelium, such as ICAM1 [41]. However, the binding activity of a highly monoclonal parasite line expressing a DC8 variant (IT4var19) [19] did not increase on any of the TNF-α activated endothelial cells (Figure 2C), which suggests that DC8 parasites are not dependent on the pro-inflammatory cytokine TNF-α for tight endothelial binding.

**Figure 4:**
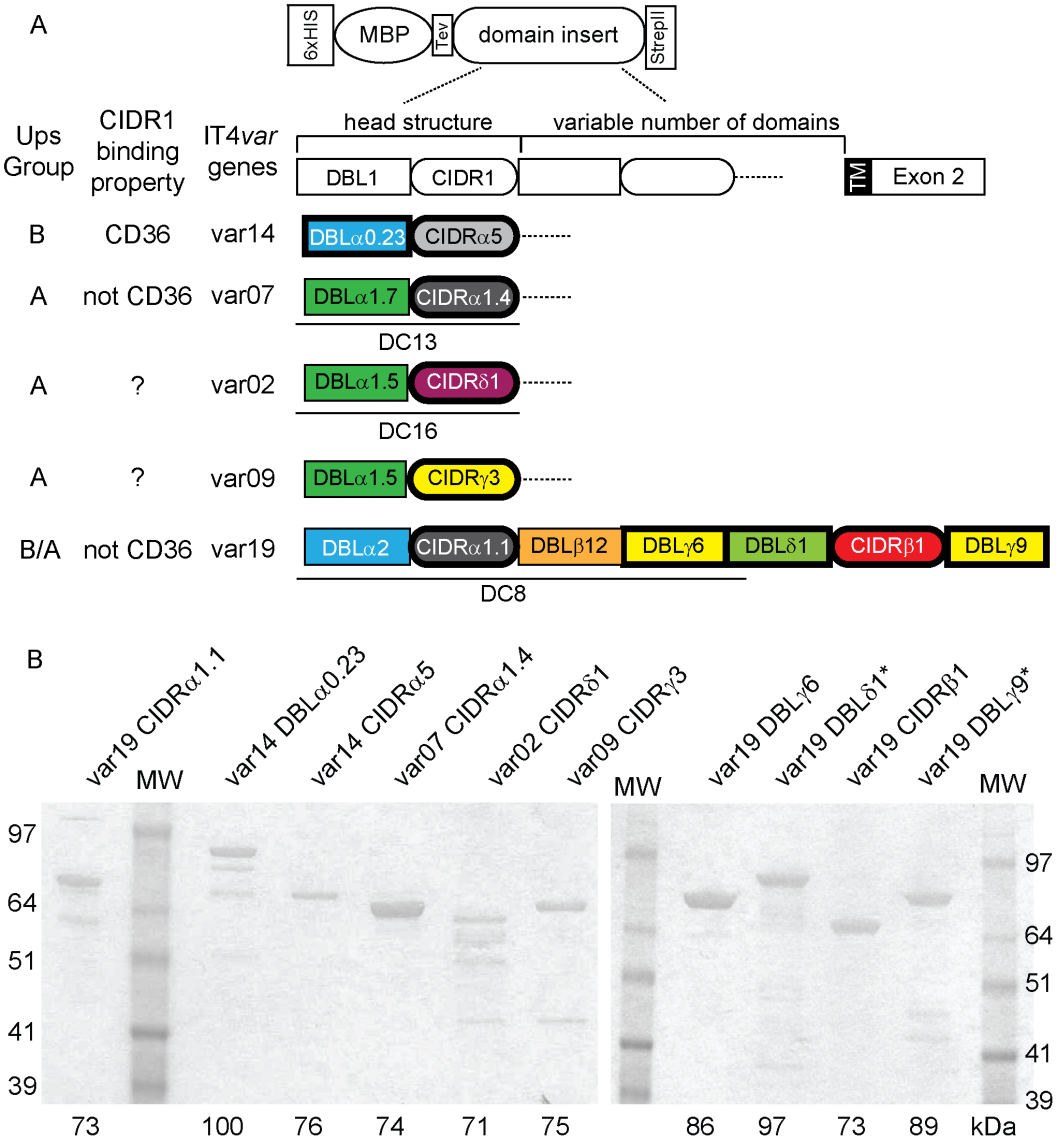
Production of PfEMP1 adhesion domains. (A) Schematic of the MBP-fusion protein constructs and a general PfEMP1 protein domain architecture. The complete extracellular domain architecture is illustrated for IT4var19 and the head structure for the other proteins. Tandem domain arrangements associated with DC8, DC13, or DC16 cassettes are underlined [14]. PfEMP1 protein domains with bold outlines were expressed as recombinant proteins. (B) Proteins were visualized using SDS/PAGE gel and GelBlue code staining. Due to the number of samples, separate gels were used to visualize the entire panel of proteins used in this study.

IT4var19 contains five DBL domains and two CIDR domains (Figure 4A). The DC8 cassette is defined by the tandem arrangement of the first four N-terminal domains (NTS-DBLα2, CIDRα1.1, DBLβ12, and DBLγ6) [14]. We previously expressed three of the four DC8 domains and found that NTS-DBLα2, CIDRα1.1 and DBLβ12 bind brain endothelial cells [19]. Here, we expressed the remaining four domains in the IT4var19 protein (DBLγ6, DBLδ1, CIDRβ1, and DBLγ9) as His-MBP-strepII fusion proteins (Figure 4A) to investigate whether any of the seven IT4var19 domains discriminated between different endothelial cell types. As controls, we generated recombinant proteins consisting of 6-His-MBP alone, and a 6-His-MBP- IT4var14 NTS-DBLα0.23-strepII construct from the CD36- and ICAM1-binding A4ultra parasite [39]. All of the recombinant proteins migrated at the expected molecular weight in SDS/PAGE gel (Figure 4B). We were able to produce all but two of the proteins at yields of 100 µg/L or more. The only exceptions were DBLδ1 and DBLγ9, which we were only able to produce at substantially lower yields (<100 µg/L).

**Figure 5:**
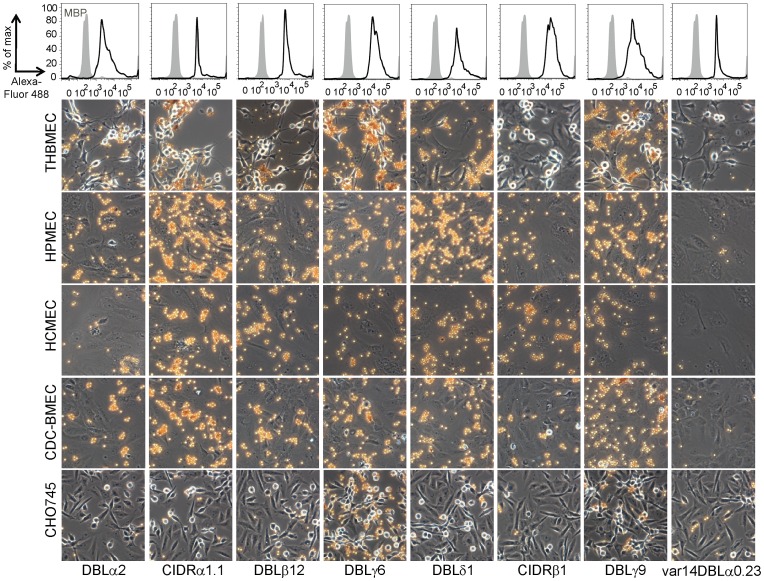
Multiple domains in the DC8-IT4var19 variant exhibit widespread endothelial binding activity. Top panel: The coupling of recombinant protein to Dynal beads was quantified by flow cytometry with anti-StrepII antibodies; MBP control (grey histogram), PfEMP1 domains (black histogram). The MBP protein used as a negative control lacks the StrepII tag added to the C-terminus of the PfEMP1 domains. Bottom panel: Representative images for each bead coupled PfEMP1 domain to THBMEC, HPMEC, HCMEC, CDC-BMEC, or non-endothelial control CHO-745 cell. A control PfEMP1 domain (var14DBLα0.23) is also represented.

To assess the endothelial specificity of the individual domains, the seven recombinant proteins were coated on Dynal beads and investigated for binding to the four microvascular endothelial cells; brain, heart, lung, and bone marrow. All seven DC8-IT4var19 domains bound to endothelial cells, but none of the domains bound to the control non-endothelial CHO-745 cell line, except for DBLγ6 and to a lesser extent DBLγ9 (Figure 5). In contrast, MBP-alone and IT4var14 DBLα0.23 recombinant protein did not bind to any cell line. The CIDRβ1 domain was the weakest binding domain, although it had slightly better binding to HCMEC than other endothelial cells. Both DBLα1 and DBLβ12 domains showed moderate binding to all four endothelial cells and CIDRα1.1, DBLγ6, DBLδ1 and DBLγ9 domains displayed high binding activity across all endothelial cell types (Figure 5).

In order to ensure that differences in the observed binding levels were due to differences in the adhesive properties of the endothelial domains, rather than artifacts generated by variations in the quantity of recombinant protein coupled to the beads, the coated beads were analyzed by flow cytometry using antibodies against the C-terminal strepII tag in the recombinant proteins. We found that all of the recombinant proteins were equally well coupled to the beads (top panel, Figure 5).

**Figure 6:**
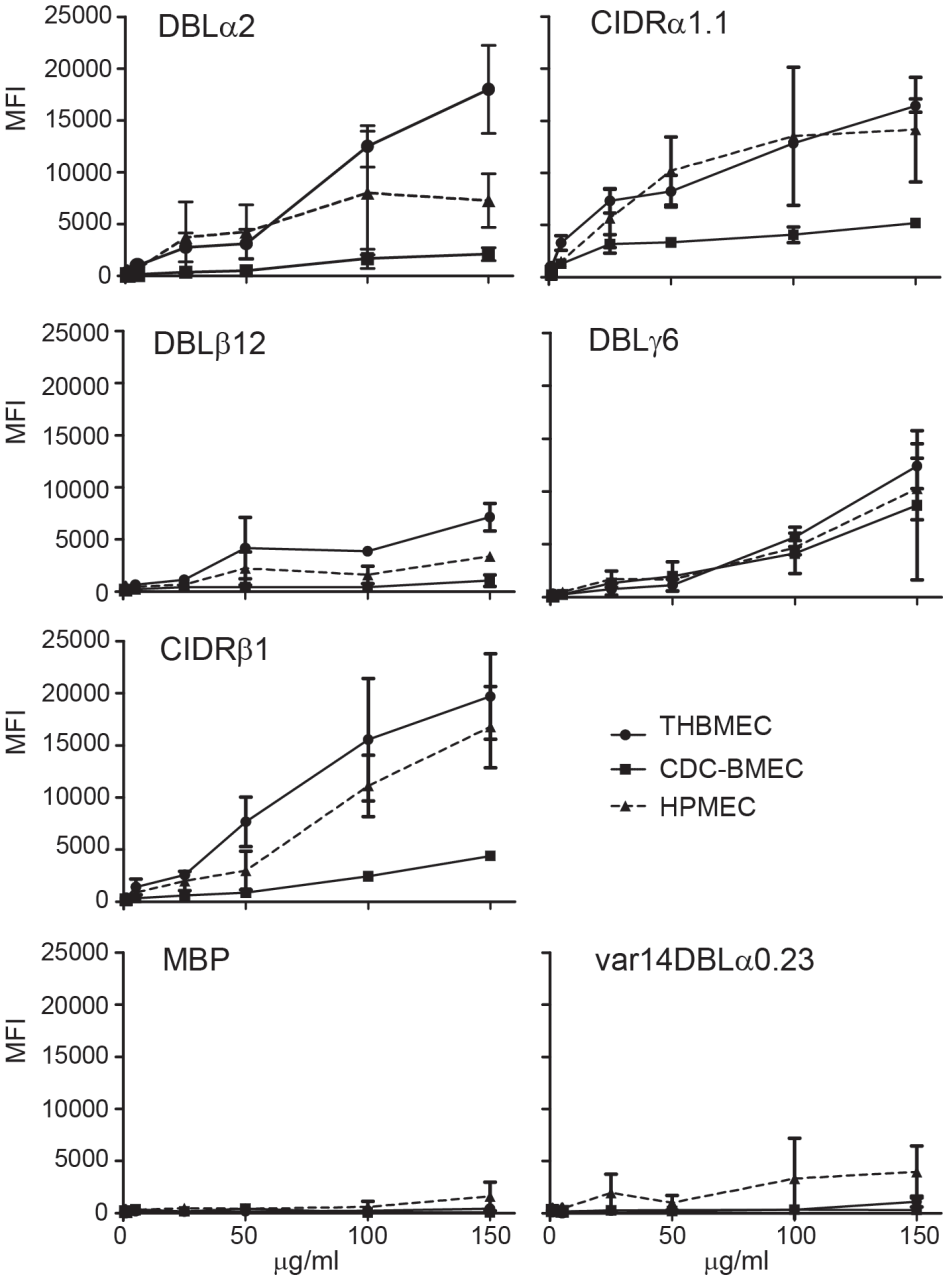
Dose-dependent binding of IT4var19 adhesion domains to endothelial cells. Binding of recombinant proteins to THBMEC, HPMEC, and CDC-BMEC was assessed by flow cytometry using anti-StrepII tag antibodies.

To further investigate the endothelial specificity of different domains, the five IT4var19 domains produced at higher yield were also evaluated in a flow cytometry assay. In this assay, endothelial cells were lifted from tissue culture dishes and then incubated with recombinant proteins. All five recombinant proteins bound in a dose-dependent fashion to THBMEC, HPMEC, and CDC-BMEC, but there was little or no binding of the negative control proteins (Figure 6). In this assay, DBLα2, CIDRα1.1, and CIDRβ1 had the highest binding activity to brain and lung endothelial cells, DBLγ6 had moderate activity, and DBLβ12 had lower activity. The higher binding activity of the soluble CIDRβ1 domain compared to the CIDRβ1-coated beads could be due to steric hindrance. Taken together, all seven extracellular domains in DC8-IT4var19 encoded endothelial binding activity and most domains did not discriminate between endothelial cell types, although individual domains did exhibit differences in the level with which they bound to particular cell types.

### The interaction of IT4var19 domains with endothelial receptors differ in protease sensitivity

**Figure 7:**
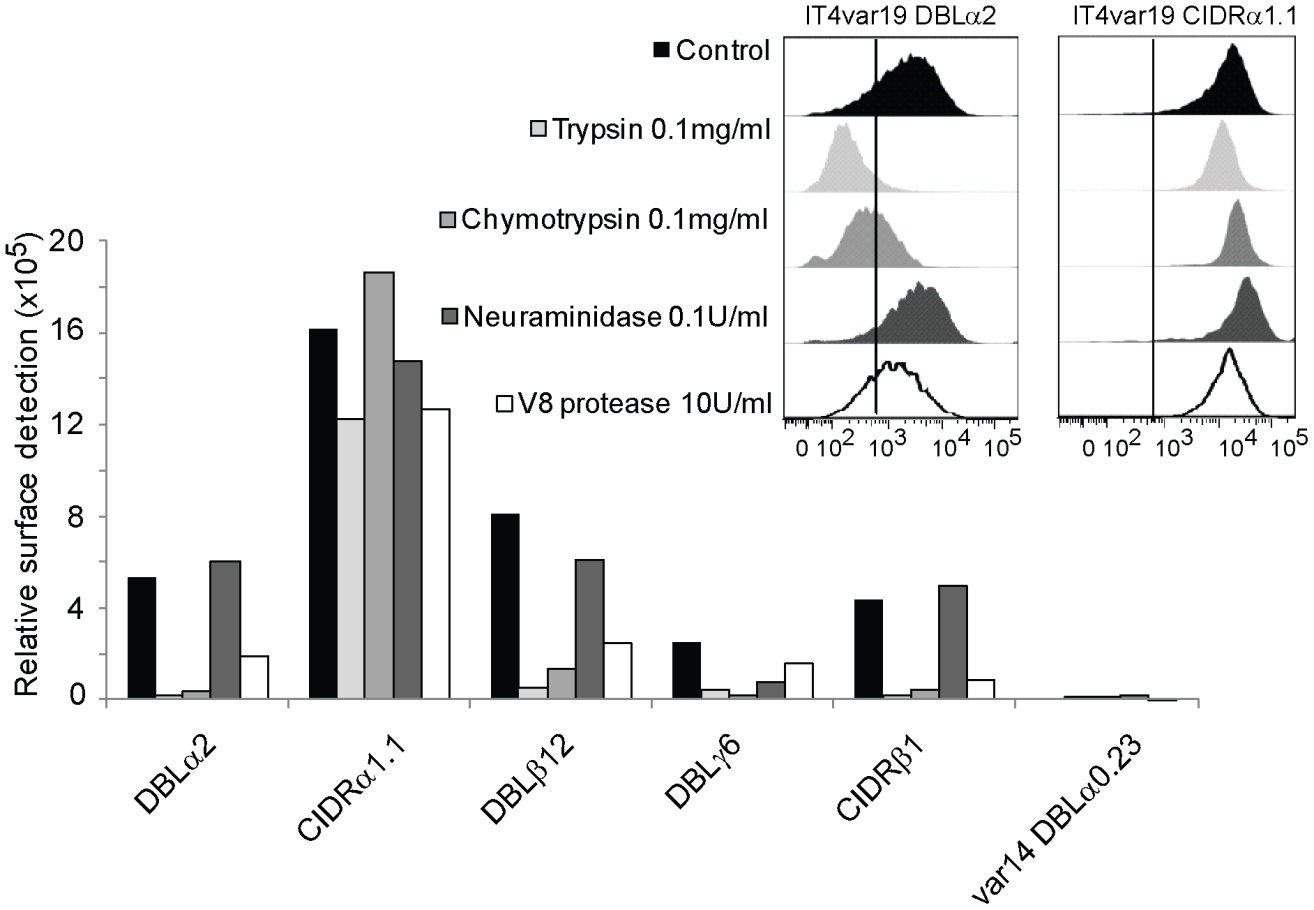
IT4var19 domains display distinct protease-sensitive binding profiles for brain endothelial cells. Binding of DC8-var19 recombinant protein to THBMEC was assessed by flow cytometry via anti-StrepII tag antibodies on THBMEC cells pretreated with trypsin, chymotrypsin, neuraminidase or V8 protease. Inset shows example histograms. Results are expressed as relative surface detection ( = proportion of cells antibody reactive × MFI of reactive cells).

To investigate the host receptor specificity of DC8-IT4var19 domains, THBMEC were pre-treated with different proteases or glycanases before assessing their capacity to bind the recombinant proteins by flow cytometry (Figure 7). Six of the seven DC8-IT4var19 domains recognized trypsin, chymotrypsin, and V8 protease sensitive brain receptors suggesting they bound to a proteinaceous receptor (Figure 7). Of the six, the DBLα2, DBLβ12, and CIDRβ1 had similar protease sensitivities. In contrast, adhesion of CIDRα1.1 was not affected when THBMEC were pretreated with the three proteases, although there was a slight decrease of CIDRα1.1 binding to THBMEC after treatment with ten-fold higher concentrations of trypsin and chymotrypsin (1 mg/ml). However, protease treatment at these concentrations also degraded the THBMEC membranes, which resulted in much higher background (data not shown). Of interest, none of the IT4var19 domains were sensitive to neuraminidase pretreatment, except for DBLγ6. This is the only domain that bound to CHO-745 cells and may interact with a sialoglycoprotein. Based on the differential protease sensitivities, we hypothesize there may be at least three host receptor specificities encoded by the intact IT4var19 PfEMP1 protein.

### DC8 and DC13 CIDR1 domains exhibit widespread endothelial binding activity

**Figure 8:**
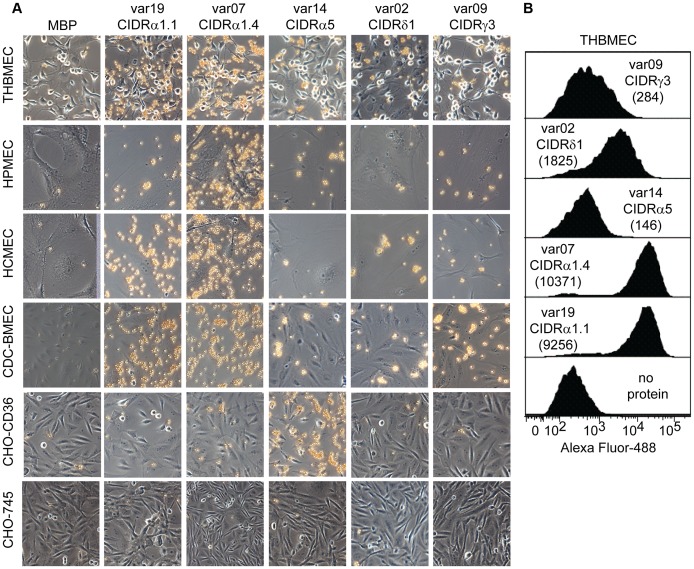
Binding of CIDR1 domains present in the semi-conserved head structures of DC8/DC13 or other UpsA PfEMP1 variants. (A) Representative images for bead coupled CIDR domain binding to endothelial cells (THBMEC, HPMEC, HCMEC, CDC-BMEC), CHO-CD36, or CHO-745 cells. (B) Surface staining of each recombinant CIDR domain to live THBMEC determined by FACS analysis. Normalized MFI indicated inside parenthesis.

The CIDR1 domain in the PfEMP1 semi-conserved head structure plays a key role in infected erythrocyte binding. This domain has diverged into different sequence types (α1, α2-6, β, γ, or δ) that differ in CD36 binding activity [14]. Group B and C PfEMP1 variants encode CIDR α2-6 sequence types that usually bind CD36, while group A and B/A encode other CIDR sequence types (α1, β, γ, or δ) that do not bind CD36 [18]. To investigate binding selection on the group A protein head structure, we produced representative CIDR1 recombinant proteins (α1, γ, and δ sequence types) (Figure 4) and compared them for binding to different endothelial cells with a control CD36-binding domain from IT4var14 (CIDRα5). In the coated bead binding assay, var19 CIDRα1.1 (DC8) and var7 CIDRα1.4 (DC13) exhibited strong and dispersed binding to all four endothelial cell types (Figure 8A). By comparison, var14 CIDRα5, var02 CIDRδ1, and var09 CIDRγ3 all displayed low and patchy binding to different endothelial cells types. Likewise, in a flow cytometry binding assay with THBMEC, var19 CIDRα1.1 (DC8) and var7 CIDRα1.4 (DC13) bound at high levels, var02 CIDRδ1 bound at low levels, and var09 CIDRγ3 and var14 CIDRα5 bound at very low or negligible levels (Figure 8B). As expected, the positive control var14 CIDRα5 domain from group B was the only one that bound to CHO-CD36 cells, indicating group A CIDR1 domains employed non-CD36 binding interactions.

We previously showed that 100 µg/ml IT4var19 CIDRα1.1 domain could partially inhibit IT4var19-IE binding to brain endothelial cells by ∼50%, but not the DBLα2 or DBLβ12 domains [19]. When we tested the four new DC8-IT4var19 recombinant proteins, none of the individual domains at 100 µg/ml or mixtures of the four domains (total concentration = 100 µg/ml) inhibited IT4var19-IE binding to THBMEC, but again the CIDRα1.1 domain on its own inhibited binding by ∼40–50% (Figure S4). In addition, the var7 CIDRα1.4 (DC13) strongly cross-inhibited IT4var19-IE binding to brain endothelial cells (∼80–90%, Figure S4), while the CD36-binding var14 CIDRα5 domain did not. The strong cross-inhibitory activity of var7 CIDRα1.4 could be because it appeared to have slightly higher endothelial binding activity than var19 CIDRα1.1 in the coated bead and flow cytometry binding assays (Figure 8). Taken together, these findings suggest that CIDRα1.1 and α1.4 subtypes in the PfEMP1 head structure engage in a key binding interaction with brain endothelial cells and that DC8 and DC13 CIDR1 domains may bind the same receptor.

## Discussion

Despite the importance of PfEMP1 as a virulence factor, relatively little is known about how PfEMP1 binding specificity may influence parasite tropism for tissue sites other than brain and placenta tissue [19], [21], [27], [42]. It has been hypothesized that the adhesion traits encoded by UpsA PfEMP1 variants may confer a selective advantage on parasites displaying these proteins in malaria naïve hosts [43], [44]. However, this concept has received limited experimental investigation. One of the best studied adhesion receptors is CD36 [35], [36]. This adhesion property is encoded into most or all group B and group C PfEMP1 proteins [17], [18], but it is not associated with group A and B/A PfEMP1 variants, which tend to be expressed in individuals with limited malaria immunity and have been associated with more severe infections [20], [22]–[25]. As CD36 expression differs between microvascular sequestration sites [41], it is possible that diversification of PfEMP1 proteins into CD36 binding and non-CD36 binding variants could be shaped by within-host competition for binding niches and may be a strategy to maximize parasite survival and transmission.

In this study, we demonstrate that non-CD36 binding DC8 and DC13 variants were the major *var* transcripts selected on diverse microvascular endothelial cell types, including those that mostly lack CD36 surface expression (brain, heart, bone marrow) and those containing a mixture of CD36^+^ and CD36^−^ cells (lung). Although CD36 is generally thought to be a relatively ubiquitous receptor on microvascular endothelial cells, it is not expressed on the endothelial lining of brain, placenta or bone marrow sinuses and is strongly expressed by liver, spleen, and lung endothelial cells [41], [45], [46]. Our findings suggest that the importance of non-CD36 expressing vascular beds may have been underappreciated for *P. falciparum*-IE sequestration and further indicate that DC8 and DC13 variants may even outcompete CD36 binding variants in sequestration sites with variegated CD36 levels, as observed in the lung endothelial selection. Of interest, the binding activity of a DC8-infected erythrocyte was not increased on TNF-α activated endothelial cells, although this is known to upregulate potential cytoadhesion receptors on endothelial cells, such as ICAM1 [40]. It is possible that this lack of TNF-α dependence may give DC8 variants an advantage at early stages of malaria infection before widespread endothelial activation.

Intriguingly, all seven DBL and CIDR domains in IT4var19 DC8 variant bound endothelial cells and individual PfEMP1 domains were largely not discriminatory between brain, lung, heart and bone marrow endothelial cells. Based on protease-treatments, there appear to be at least three different host receptor binding specificities on brain endothelial cells. Although the binding of individual PfEMP1 domains may not always reflect the activity of native protein, several PfEMP1 adhesion traits such as CD36, ICAM1, complement receptor 1, and others have been mapped to individual PfEMP1 domains [10]. This analysis suggests that DC8 domains do not bind the common cytoadhesion receptors CD36 and ICAM1. Future work will need to define the DC8 binding partner interactions. The redundancy of DC8 domain-receptor interactions may strengthen the IE-endothelial interaction. Indeed, most individual DC8 domains were unable to inhibit IE binding to brain endothelial cells, except for CIDRα1.1 [19]. These findings have important implications because it suggests there is likely to be competition between different microvascular sites for DC8 parasite binding depending on an array of host receptor expression levels. Furthermore, it is possible that non-brain microvascular sites may be reservoirs for cerebral binding variants and anti-adhesion strategies focused on individual domains may only be partially effective.

The CIDR1 domain in the PfMEP1 protein head structure plays a key role in IE binding and has diverged into CD36 binding and non-binding types [17], [18]. Our findings confirm that group A CIDR1 domains are not under strong selection to bind CD36 and suggest that DC8 and DC13 CIDR1 domains bind a common receptor with widespread endothelial distribution. By comparison, two other Group A head structures (CIDRγ and δ) bound at low or negligible levels to all of the endothelial cells tested. CIDRγ and CIDRδ are frequently found in PfEMP1 variants that bind red blood cells and facilitate infected erythrocyte rosetting with uninfected erythrocytes [47]. Thus, it remains to be established if the CIDR1 domain is under strong endothelial binding selection in rosetting parasite variants.

Although DC8 and DC13 *var* gene expression is not restricted to severe malaria infections [24], it might be expected that potentially deadly adhesive traits would be at a selective disadvantage. Survival versus reproductive trade-offs is a key concept in evolutionary biology. Evolutionary theory predicts that fast growing parasites benefit from a larger pool to produce transmissible gametocytes but there is a trade-off with increasing risk of host death [48], [49]. Whereas antigenic variation is generally thought to increase transmission success by enhancing infection chronicity, specific *P. falciparum* adhesion traits such as placental binding may increase parasite transmission success by providing new transmission opportunities [50], [51]. Our findings support the hypothesis that DC8 and DC13 variants give parasites a growth and transmission advantage and could also explain why they tend to be expressed in early childhood infections, before children in malaria-endemic areas have acquired antibodies that can effectively counter this set of PfEMP1 proteins. In a related finding that is also of interest, bone marrow aspirates from patients with cerebral malaria showed IEs adhering within bone marrow sinuses [52]. Bone marrow sinuses offer close proximity to reticulocytes that are preferentially invaded by *P. falciparum* [53] and it is also the major site of gametocyte sequestration [54]. It will be interesting to investigate if these traits are linked, such that bone marrow sequestration promotes red blood cell invasion efficiency or sexual conversion efficiency.

In conclusion, this study suggests that the severe malaria associated DC8 variants are under selection to bind to diverse microvascular endothelial cells via multiple domain-receptor interactions. This avid binding trait may enhance parasite growth and confer a transmission advantage sufficient to keep these potentially deadly adhesion traits in the *var* gene repertoire, despite their association with relatively rare instances of severe disease in immunonaïve hosts.

## Materials and Methods

### Ethics statement

Human blood for parasite culture was collected as part of the Seattle Biomedical Research Institute Blood Draw Protocol (HS013, WIRB Protocol # 20041115, approved by Western Institution Review Board). Blood was collected specifically for this study and written informed consent was obtained from all donors.

### Parasite

*P. falciparum* parasites were cultured under standard conditions using human O red blood cells in RPMI-1640 medium (Invitrogen) supplemented with 10% pooled human A+ serum. ItG-ICAM-1 and A4long parasite lines were derived from IT4/25/5 strain by selecting on ICAM1 [55] or limited dilution cloning [8], respectively. All parasites lines were synchronized by treatment with 5% sorbitol and were routinely enriched by gelatin flotation to ensure IEs maintained their “knob-like” adhesion complexes [56].

### Human microvascular endothelial cell cultures

Immortalized HBMEC were derived by simian virus 40 large T antigen (SV40-LT) transformation (THBMEC) as previously described [57]. THBMEC were cultured in growth media supplemented by geneticin G418 (100 µg/ml) instead of the growth factor ECGS [19]. Primary human pulmonary (HPMEC #3000) and cardiac microvasculature endothelial cells (HCMEC #6000) were purchased from ScienCell and cultured following the manufacture's recommendations in fibronectin coated flasks or slides. HPMEC and HCMEC were used within 6 passages. Human bone marrow endothelial line, CDC-BMEC, was kindly provided by Dr. Kathryn Kellar (CDC, USA) [45]. CDC-BMEC were cultured in MCDB 131 Formula (# 10372019, Invitrogen) supplemented with 10 mM/L L-Glutamine, 10 ng/ml mouse epidermal growth factor, (#354001, Becton-Dickinson), 1 µg/ml hydrocortisone (H0888, Sigma) and 10% fetal bovine serum. CDC-BMEC were grown on collagen coated flasks or slides.

### Immunofluorescence and flow cytometry analysis

Immunofluorescence assays were performed on confluent monolayers of cells as previously described [19]. In brief, anti-vWF/Factor VIII (1/40, Dako A0082) antibodies were added to methanol fixed cells and the remaining antibody labelings were performed on live cells. The following mouse monoclonal antibodies were used: anti-ICAM1-PE conjugated (1 µg/ml, Abcam, ab19756), anti-VCAM1/CD106-PE conjugated (10 µl for 10^5^cells, R&D Systems, FAB5649P), anti-CD36 (clone FA6-152, 2 µg/ml, Beckman Coulter Immunotech), anti-E-selectin (10 µg/ml, ELAM1, R&D Systems, BBA16), anti-CD31-PE conjugated (PECAM) (1/50 dilution, Molecular Probes), and anti-ICAM2-FITC conjugated (2 µg/ml, clone CBR-IC2/2, Abcam, ab27559). Mouse monoclonal antibodies were revealed by goat anti-mouse Alexa488 coupled antibodies (Molecular Probes, 1/200) for 30 min on ice followed by a tertiary rabbit anti-goat Alexa488 coupled antibodies (Molecular Probes, 1/500). Goat anti-human CX3CL1/Fractalkine (15 µg/ml, R&D Systems AF365) antibodies were detected with rabbit anti-goat Alexa488 coupled antibodies (Molecular Probes, 1/500). Cell nuclei were stained with DAPI (Sigma, 25 µg/ml). Prior to visualization, cells were fixed with 2% w/v of paraformaldehyde for 10 min before mixing with Prolong Antifade kit (P7481, Molecular Probes). Analyses were done on a Nikon Eclipse E600 upright microscope with a Cool Snap CCD camera and Metamorph software.

For flow cytometry, a T75 cm^2^ flask of confluent human endothelial cell culture was lifted with 10 mM EDTA and 50,000 cells were distributed in 96 well plates. Antibody staining was done as described above. Single live cells were gated using Live/Dead fixable violet Dead cell stain kit (Molecular Probes). Cells were fixed with 2% w/v of paraformaldehyde for 10 min and 20,000 cells were counted in an LSRII (Becton Dickinson). Results were analyzed using FLOWJO 8.1 software (Tree Star Inc.).

### Selection of infected erythrocytes on human endothelial cells

IE selections were done as previously described [19]. In brief, gelatin-enriched, trophozoite stage A4long or ItG-ICAM1 parasite cultures were added to confluent monolayers of endothelial cells in coated T25 cm^2^ flasks. The IE suspension was resuspended every 15 min by gently rocking the flask during the 1 hr incubation at 37°C. Unbound erythrocytes were removed by rocking the flasks back and forth. Between washes, the level of binding was assessed by checking under an inverted microscope. After the last wash, the binding medium was removed and 5 ml of complete *Plasmodium* culture medium were added with 250 µl of 50% hematocrit. The endothelial cell-bound IE flask was then incubated overnight at 37°C in parasite gas mixture. The next day, red blood cells were resuspended and transferred to a new flask. The selection was repeated twice after allowing 3 to 4 cycles of parasite growth between pannings.

### Determination of *var* transcription by Q-RT-PCR

The *var* gene transcription profiles were performed using the same set of primers and PCR conditions as previously described [17]. In brief, RNA was extracted in Trizol LS (Invitrogen) from ring stage parasites at ∼6–12 hrs post-invasion. Quantitative Real-time PCR reactions were performed on an ABI Prism 7500 thermocycler and relative transcription was determined by normalization to the control housekeeping gene adenylosuccinate lyase (ASL, PFB0295w). The result was expressed as percentage of individual *var* transcript relative to the total transcription of the 56 IT4 *var* transcripts.

### Binding and binding inhibition assays of *P. falciparum*-IEs to human endothelial cells

THBMEC, HCMEC, HPMEC and CDC-BMEC were seeded on coated 8 well slides (BD Biocoat) 3 to 4 days before assays and allowed to grow to confluency. Binding assays and washes were performed with pre-warmed binding medium (RPMI-1640 medium containing 0.5% bovine serum albumin, pH 7.2). IEs were gelatin-enriched and resuspended to 5×10^6^ IEs/ml solution and then were added to a multiwell slide. For TNF-α stimulation, 10 ng/ml of TNF-α (Sigma, T0157) was added to endothelial cell monolayers 24 hrs before the binding assay. For antibody binding inhibition assays, endothelial cells were pre-incubated with mouse monoclonal anti-human CD36 (clone FA6-152, 5 µg/ml, Beckman Coulter Immunotech) or mouse monoclonal anti-human ICAM1 (clone 15.2, 5 µg/ml Abcam ab20). For protein binding inhibition assays, the THBMEC monolayer was pre-incubated for 30 min at 37°C with 100 µg/ml or a mixture of 200 µg/ml of recombinant proteins prior adding 100 µl of IEs suspension. After 30 min to 1 hr incubation at 37°C, non-binding erythrocytes were removed by inverting the slide into a 37°C warmed bath of binding medium for 10 min to let the unbound erythrocytes fall by gravity. Slides were then fixed in 1% glutaraldehyde for 30 min at room temperature and stained with Giemsa for 5 min. Binding was quantified by determining the number of IEs adhering per mm^2^ of endothelial cells in 10 random fields under 400× magnification. All binding assays were done at least in duplicate.

### Parasite growth curves

For parasite growth curves, parasite cultures were diluted to 0.1% ring-stage parasites and grown for two growth cycles, followed by dilution to 0.1% ring-stage parasites and growth over two additional cycles. Parasitemia was monitored daily by counting the percentage of IEs in Giemsa-stained blood smears over 5000 erythrocytes.

### Production of DC8-*var19* encoded recombinant proteins

Recombinant proteins (His_6_-MBP-TEV-PfEMP1 insert-StrepII-tagged) were produced in pSHuffle Express (NEB) expression hosts. Inserts were cloned using Gateway destination vector technique as previously described [19]. A TEV cleavage site was added between the MBP fusion protein and the domain of interest. Amino acid boundaries were the following for IT4var19 (gene accession number EF158075) (numbering starts from the first methionine in the protein): NTS-DBLα2 (M1-V484), CIDRα1.1 (C485-C732), DBLβ12 (P733-C1220), DBLγ6 (C1277-C1630), DBLδ1 (C1722-C2186), CIDRβ1 (S2187-C2439) and DBLγ9 (C2525-C2903). For the other proteins constructs, we used the following boundaries IT4var14 NTS-DBLα0.23 (M1-C484), IT4var14 CIDRα5 (W486-E755), IT4var07 CIDRα1.4 (P467-C717), IT4var02 CIDRδ1 (P458-C713), and IT4var09 CIDRγ3 (P464-C721). Fusion protein production was induced using 0.5 mM IPTG (EMD) at 20°C (225 rpm) for 14–16 hrs. After induction, pSHuffle cultures were harvested, frozen, resuspended in lysis buffer and then were subjected to several rounds of sonication (Sonics & Materials VCX-600 Vibra-Cell, 35% amplitude, 30S, 0.5S pulse-pause intervals) until the suspension had substantially clarified. Protein purification was done in two steps, first through Ni-NTA agarose resin (GE) and eluted with 500 mM immidazole (Sigma). Then the eluate was diluted threefold to reduce the imidazole concentration and purified through a Strep-Tactin Superflow resin (Qiagen) and eluted with lysis buffer bearing 2.5 mM Desthiobiotin (Sigma). The eluate was dialyzed into cold 1× PBS and quantitated via BCA assay (Pierce) before visualization on 4–12% Bis-Tris SDS-PAGE acrylamide gels (Invitrogen) and GelCode Blue staining (Thermo). Purified fusion protein was stored at −80°C until use.

### Binding of PfEMP1 domain-coated Dynal beads to human endothelial cells

For the coated bead binding assay, recombinant proteins were coated onto 10^7^ sheep anti-mouse IgG coated Dynal beads (Invitrogen, 110.31) using 1.5 µl of a mouse anti-MBP monoclonal antibody diluted into PBS/0.1% BSA at 1 µg/µl (NEB, E8032S) for 1 hr under rotating agitation. After two washes, antibody-coupled beads were incubated with His-MBP-insert-StrepII fusion protein or His-MBP protein for 1–2 hrs, followed by two washes. The extent of recombinant protein binding to beads was analyzed by labeling beads with rabbit polyclonal anti-StrepII tag antibodies (1/40, Genscript, NWSHPQFEK antibody, A00626) and goat anti-rabbit Alexa488 coupled antibodies (Molecular Probes, 1/500) and detecting by flow cytometry in an LSRII (Becton Dickinson) and FLOWJO 8.1 software (Tree Star Inc.). For the binding assay, THBMEC, HPMEC, HCMEC, CDC-BMEC, CHO-CD36, or negative control CHO-745 cells were grown on coated 8-well slides. One million protein coupled Dynal beads were resuspended in 0.3 ml of binding medium (RPMI-1640 medium containing 0.5% bovine serum albumin, pH 6.8) and added to each monolayer of cells per well. After 1 hr incubation at 37°C, each slide was inverted into a 37°C warmed bath of binding medium for 10 min to let the unbound beads fall by gravity. Slides were then fixed with 1% glutaraldehyde for 30 min. The number of beads adhering per endothelial cells was evaluated in random fields under 200× magnification and pictures were captured using the Qcapture software.

For flow cytometry binding analysis with soluble PfEMP1 recombinant proteins, endothelial cells were lifted by 10 mM EDTA and 5×10^5^cells/ml were incubated with different concentrations of PfEMP1 recombinant proteins for 30 min on ice. After washing with 1× PBS, recombinant protein binding was detected by labeling with rabbit polyclonal anti-StrepII tag antibodies followed by goat anti-rabbit Alexa488 coupled antibodies (Molecular Probes). Flow cytometry was done as described above.

### Binding of DC8-*var19* recombinant proteins to THBMEC after protease digestion

For each protease treatment, one T25 cm^2^ flask of THBMEC was seeded to reach confluency on the day of experiment. Cells were first washed once with 1× PBS. Then, either 2 ml of Trypsin TPCK (0.1 mg/ml, Sigma T1426) or Chymotrypsin TLCK (0.1 mg/ml, Sigma C3142) was applied for 15 min at 37°C. Digestion was stopped by adding 2 ml Trypsin-Chymotrypsin inhibitor (Soybean, 1 mg/ml, Sigma T9777) for 5 min. For neuraminidase digestion, 2 ml at 0.1 unit/ml was incubated with the cell monolayer for 30 min at 37°C before stopping the reaction with THBMEC media for 5 min. For the V8 protease digestion, a T25 cm^2^ of THBMEC cells were lifted with 10 mM EDTA and pelleted. Cells were resuspended into 0.5 ml V8 protease solution (10 unit/ml, endoproteinase Glu-C, Roche #10791156001) for 5 min at 37°C. V8 protease digestion was stopped by adding THBMEC media and cells were washed with washing buffer. Flow cytometry was done as described above.

### Statistical analyses

Statistical analyses were performed with Prism Software. Data were analyzed by Krustal-Wallis one-way Anova test. Results are expressed as means ± standard deviations.

## Supporting Information

Figure S1 **Receptor expression profiles of endothelial cells.** Expression of the endothelial cell markers and potential parasite cytoadhesion receptors were analyzed by immunofluorescence assay. The presence of the endothelial marker, VWF, was done on methanol fixed cells to detect the protein in Weibel-Palade bodies. The remaining analyses were performed on live cells. Three endothelial cell types, HPMEC (pulmonary), HCMEC (cardiac), and CDC-BMEC (bone marrow) were analyzed for each receptor.
(PDF)

Figure S2 **Binding levels of** ***P. falciparum*** **-infected erythrocytes increase after endothelial cell selection.** The binding levels of *P. falciparum*-infected erythrocytes were compared between initial parasite cultures and parasites panned three times on the respective endothelial cells. IT4var19 (DC8 variant) and IT4var31 (CD36 binder) are highly clonal parasite lines that primarily transcribe a single *var* gene [19].
(PDF)

Figure S3 **Growth curve of clonal** ***P. falciparum*** **parasite lines.** The growth rates of two clonal parasite lines expressing IT4var19 (DC8) and IT4var31 (CD36 binder) were compared to their parental line (ItG-ICAM-1 initial). Parasite cultures were started at 0.1% ring-stage parasites and maintained for 2 cycles without dilution. On day 5, each parasite line was diluted to 0.1% ring-stage parasites and allowed to grow for 2 more cycles.
(PDF)

Figure S4 **DC8 and DC13 CIDR1 domains inhibit DC8-IE binding to THBMEC.** Infected erythrocytes expressing IT4var19 were added to a monolayer of transformed human brain endothelial cells in the presence of individual recombinant proteins or protein mixtures. (A) Binding inhibition was assessed in the presence of individual IT4var19 domains (100 µg/ml) or domain mixtures (total = 100 µg/ml). Mix 1 corresponds to an equal mixture of DBLα1, CIDRα1.1, DBLβ12, and DBLγ6. Mix 2 corresponds to the same four recombinant proteins plus CIDRβ1. Mix 3 corresponds to the same five recombinant proteins plus DBLγ9. Binding inhibition is relative to the control var14 DBLα0.23. (B) Binding inhibition was assessed in the presence of 100 µg/ml CIDR recombinant proteins. Binding inhibition is relative to the control His-MBP protein fusion.
(PDF)

Table S1 **Surface expression of receptors on different endothelial cells.**
(PDF)
