# Supplementary figures and images for: DC8 and DC13 var Genes Associated with Severe Malaria Bind Avidly to Diverse Endothelial Cells
Source: PLoS Pathog. 2013 Jun 27;9(6):e1003430. doi: 10.1371/journal.ppat.1003430 (PMC3694856; doi:10.1371/journal.ppat.1003430)

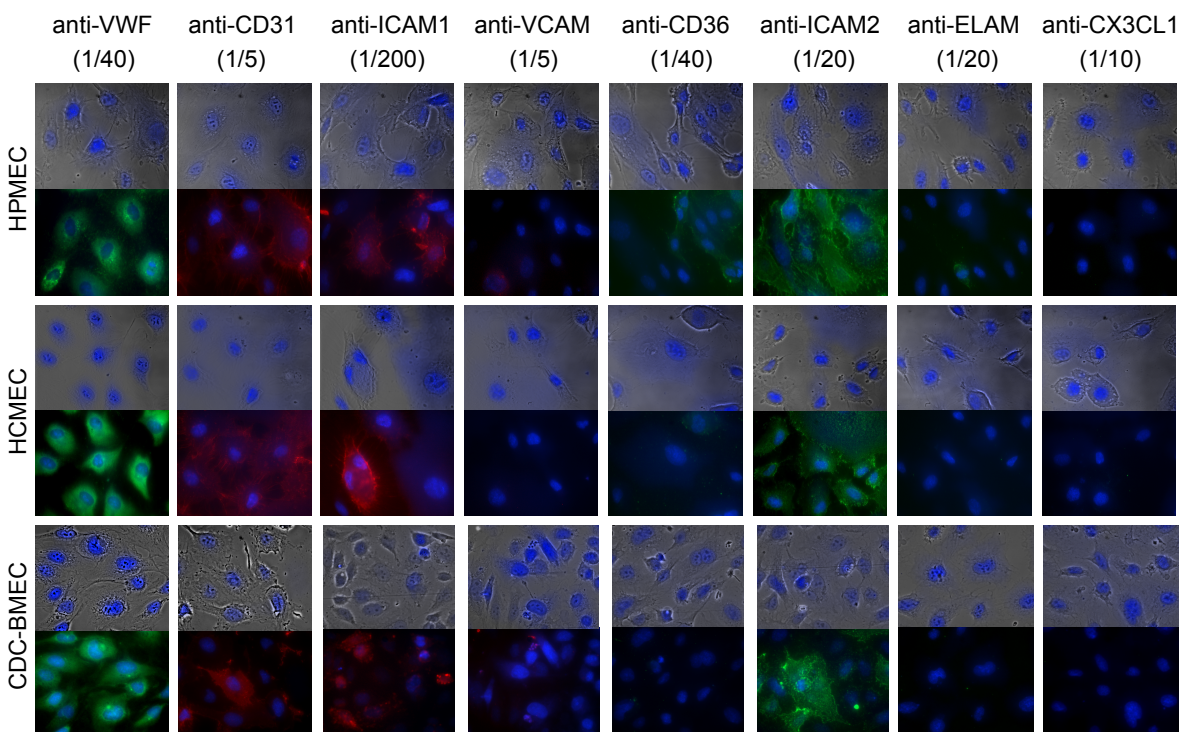

**Figure S1. Receptor expression profiles of endothelial cells.**

Supplement: Figure S1 — Receptor expression profiles of endothelial cells. Expression of the endothelial cell markers and potential parasite cytoadhesion receptors were analyzed by immunofluorescence assay. The presence of the endothelial marker, VWF, was done on methanol fixed cells to detect the protein in Weibel-Palade bodies. The remaining analyses were performed on live cells. Three endothelial cell types, HPMEC (pulmonary), HCMEC (cardiac), and CDC-BMEC (bone marrow) were analyzed for each receptor. (PDF) [file ppat.1003430.s001.pdf]

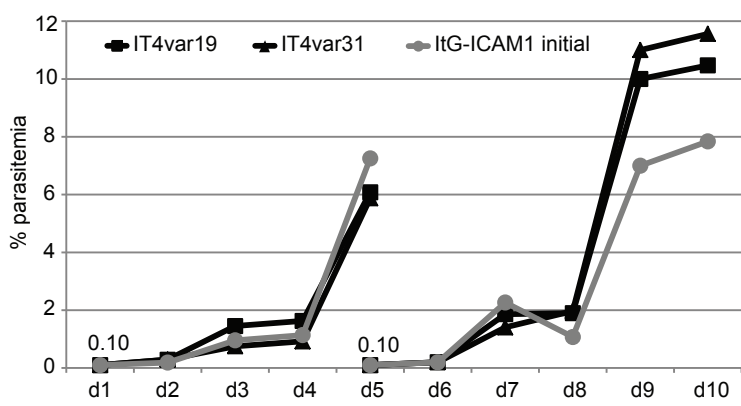

**Figure S3.** Growth curve of clonal *P. falciparum* parasite lines.

Supplement: Figure S3 — Growth curve of clonal P. falciparum parasite lines. The growth rates of two clonal parasite lines expressing IT4var19 (DC8) and IT4var31 (CD36 binder) were compared to their parental line (ItG-ICAM-1 initial). Parasite cultures were started at 0.1% ring-stage parasites and maintained for 2 cycles without dilution. On day 5, each parasite line was diluted to 0.1% ring-stage parasites and allowed to grow for 2 more cycles. (PDF) [file ppat.1003430.s003.pdf]

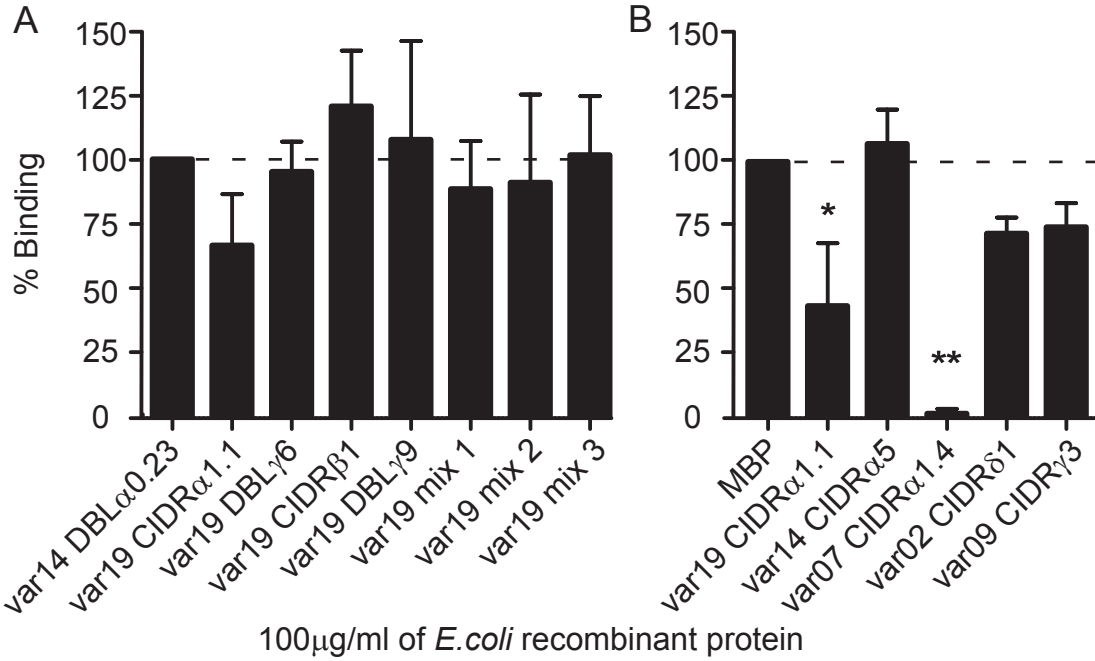

**Figure S4. DC8 and DC13 CIDR1 domains inhibit DC8-IE binding to THBMEC.**

Supplement: Figure S4 — DC8 and DC13 CIDR1 domains inhibit DC8-IE binding to THBMEC. Infected erythrocytes expressing IT4var19 were added to a monolayer of transformed human brain endothelial cells in the presence of individual recombinant proteins or protein mixtures. (A) Binding inhibition was assessed in the presence of individual IT4var19 domains (100 µg/ml) or domain mixtures (total = 100 µg/ml). Mix 1 corresponds to an equal mixture of DBLα1, CIDRα1.1, DBLβ12, and DBLγ6. Mix 2 corresponds to the same four recombinant proteins plus CIDRβ1. Mix 3 corresponds to the same five recombinant proteins plus DBLγ9. Binding inhibition is relative to the control var14 DBLα0.23. (B) Binding inhibition was assessed in the presence of 100 µg/ml CIDR recombinant proteins. Binding inhibition is relative to the control His-MBP protein fusion. (PDF) [file ppat.1003430.s004.pdf]
