# Supplementary material for: DC8 and DC13 var Genes Associated with Severe Malaria Bind Avidly to Diverse Endothelial Cells
Source: PLoS Pathog. 2013 Jun 27;9(6):e1003430. doi: 10.1371/journal.ppat.1003430 (PMC3694856; doi:10.1371/journal.ppat.1003430)
Supplement: Figure S2 — Binding levels of P. falciparum -infected erythrocytes increase after endothelial cell selection. The binding levels of P. falciparum-infected erythrocytes were compared between initial parasite cultures and parasites panned three times on the respective endothelial cells. IT4var19 (DC8 variant) and IT4var31 (CD36 binder) are highly clonal parasite lines that primarily transcribe a single var gene [19]. (PDF) [file ppat.1003430.s002.pdf]

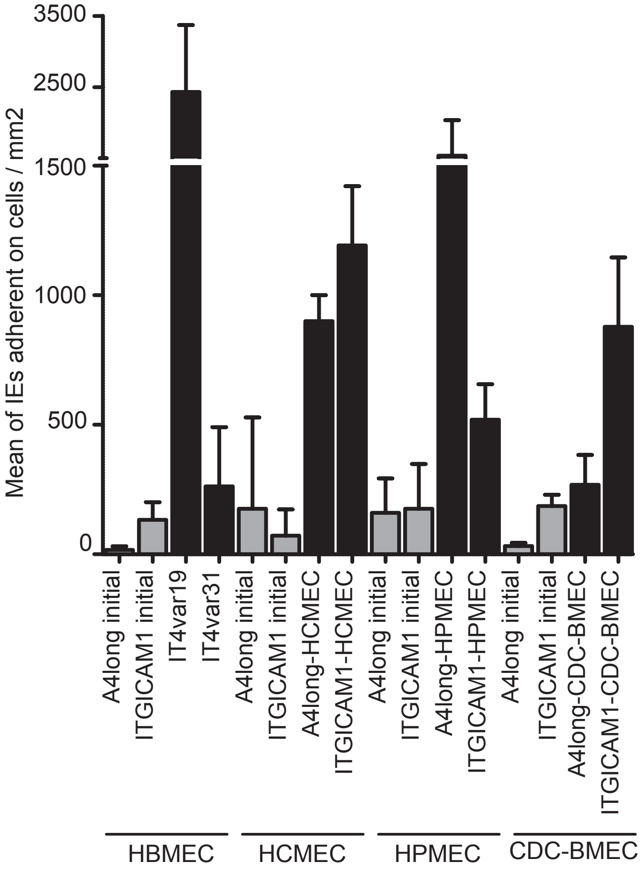

Figure S2. Binding levels of *P. falciparum*-infected erythrocytes increase after endothelial cell selection.
