# Supplementary material for: DC8 and DC13 var Genes Associated with Severe Malaria Bind Avidly to Diverse Endothelial Cells
Source: PLoS Pathog. 2013 Jun 27;9(6):e1003430. doi: 10.1371/journal.ppat.1003430 (PMC3694856; doi:10.1371/journal.ppat.1003430)
Supplement: Table S1 — Surface expression of receptors on different endothelial cells. (PDF) [file ppat.1003430.s005.pdf]

Table S1: Surface expression of receptors on different endothelial cells

	Endothelial cell marker	Endothelial adhesion molecule					
Tissue	CD31	ICAM1	VCAM	CD36	ICAM2	ELAM	CXCL1
THBMEC (brain)	7062[a] (33)[b]	492352 (98)	23668 (61)	7556 (4)	7410 (3)	1754 (1)	8204 (4)
HPMEC (pulmonary)	303072 (96)	93457 (91)	1825 (5)	115269 (33)	2704748 (97)	3777 (3)	0
HCMEC (cardiac)	266658 (98)	122608 (97)	1590 (3)	1749 (3)	1852800 (96)	1332 (3)	0
CDD-HMEC (bone marrow)	527136 (96)	10175 (25)	1374 (3)	6000 (5)	165292 (86)	3165 (5)	3900 (5)

[a] Relative surface expression level (=MFI of positive reactive cells x % positive reactive cells)

[b] % positive reactive cells
